# Hydrogen sulfide: A new therapeutic target in vascular diseases

**DOI:** 10.3389/fendo.2022.934231

**Published:** 2022-08-10

**Authors:** Cuilin Zhu, Qing Liu, Xin Li, Ran Wei, Tongtong Ge, Xiufen Zheng, Bingjin Li, Kexiang Liu, Ranji Cui

**Affiliations:** ^1^ Department of Cardiovascular Surgery, The Second Hospital of Jilin University, Changchun, China; ^2^ Jilin Provincial Key Laboratory on Molecular and Chemical Genetic, The Second Hospital of Jilin University, Changchun, China; ^3^ Department of Cardiovascular Medicine, University of Tokyo, Tokyo, Japan; ^4^ Department of Surgery, Western University, London, ON, Canada

**Keywords:** hydrogen sulfide, vascular, hypertension, angiogenesis, atherosclerosis

## Abstract

Hydrogen sulfide (H_2_S) is one of most important gas transmitters. H_2_S modulates many physiological and pathological processes such as inflammation, oxidative stress and cell apoptosis that play a critical role in vascular function. Recently, solid evidence show that H_2_S is closely associated to various vascular diseases. However, specific function of H_2_S remains unclear. Therefore, in this review we systemically summarized the role of H_2_S in vascular diseases, including hypertension, atherosclerosis, inflammation and angiogenesis. In addition, this review also outlined a novel therapeutic perspective comprising crosstalk between H_2_S and smooth muscle cell function. Therefore, this review may provide new insight inH_2_S application clinically.

## Introduction

Hydrogen sulfide (H_2_S) is recently recognized as the third gas signaling transmitter after nitric oxide (NO) and carbon monoxide (CO) despite it was once considered as toxic gas. Endogenous H_2_S production is mainly mediated by cystathionine-b-synthase (CBS), cystathionine-c-lyase (CSE), 3-mercaptopyruvate sulfur transferase (3-MST), which are the most pre-dominant enzymes of H_2_S production (1–3). Exogenous administration of H_2_S is mainly performed with NaHS salts and H_2_S related compounds.

Recent studies have proved H_2_S to be vasculoprotective by participating different cellular pathways and interfering with a variety of vascular diseases (4–7). H_2_S is endogenously produced by vascular cells or exogenously administered by H_2_S releasing donors. In the vasculature, H_2_S regulates the proliferation and migration of endothelial cells and vascular smooth muscle cell, regulates the apoptosis, oxidative stress and inflammation of vascular cells. Furthermore, H_2_S has been widely proved to regulate many vascular diseases, including hypertension, atherosclerosis (AS) and angiogenesis (8). Although the beneficial effects of H_2_S have widely been recognized, the mechanisms into the molecular pathways largely remained unknown. Deeper understanding of the working mechanisms will help put their way into further clinical application.

In this review, we review the recent findings about H_2_S in vascular diseases, including hypertension, AS and angiogenesis, as well as recent working mechanisms. The role of H_2_S will be separately discussed endogenously and exogenously here. Finally, we will discuss the possible perspectives of H_2_S in the future.

## H_2_S in hypertension

Hypertension has been a worldwide disease, accounting for 30-40% of the whole population, posing great danger to people’s health (9, 10). It is reported that H_2_S plays a role in blood pressure regulation. Despite emerging evidence from experimental studies targeting H_2_S to protect against hypertension, these results need further clinical research.

## Endogenous H_2_S in hypertension

The concentration of H_2_S in human blood has been reported within a normal range under physical conditions. However, the change of H_2_S concentration has been reported to be reduced in high blood pressure (HBP) patients, suggesting the potential regulatory role of H_2_S in HBP (11, 12). Several clinical studies have reported the relationship between H_2_S and hypertension related disorders (13). Additionally, decreased H_2_S plasmatic levels were also found in lead-induced HBP patients (14).

Furthermore, the three major generating enzymes, CSE, CBS and 3-MST were reported to be reduced in HBP patients, suggesting the endogenous synthesis of H_2_S may participate in the pathogenesis of HBP (15). Aging is an important predisposing factor for HBP. Loss of 3-MST using a genetic mouse model recuses the mouse cardiovascular system from aging-dependent disorders, thus regulating progression of HBP (16). Innate immune and adoptive immune cells are essential in the genesis and target-organ damage of hypertension. In a recent study, Cui reported that CSE-derived H_2_S promotes Treg differentiation and proliferation in an adenosine monophosphate activated protein kinase (AMPK) dependent pathway, which attenuates the vascular immune-inflammation, thereby preventing hypertension (17). Furthermore, DL-propargylgycine (PPG), a CSE inhibitor, was reported to increase BP in Wistar-Kyoto rats and to promote vascular remodeling, indicating the potential regulatory role of CSE in maintaining normal BP (18). Interestingly, in another study, treatment of Sprague-Dawley rats with CSE inhibitor, DL-propargylglycine (PAG) or CBS inhibitor, aminooxyacetic acid alone, did not alter the BP levels, while treatment with both inhibitors would significantly increase mean arterial pressure. This finding could partly explain the interaction of different H_2_S producing enzymes in regulating BP (19).

As important gas transmitters, H_2_S and NO share crosstalk in regulating pathological and physical conditions (20, 21). In hypertension, endogenous H_2_S production regulated by CSE, inhibits endogenous endothelial NO bioavailability, therefore contributing to blood pressure control (22). Sodium nitroprusside (SNP), a NO donor, was reported to increase H_2_S production *via* upregulating the CSE or CBS activity, suggesting the crosstalk between the endogenous production of two gases (23). However, the details of two gases interaction regulating blood pressure remain to be elucidated.

Taken together, endogenous H_2_S production acts as an important physiological mediator that regulates BP homeostasis and H_2_S deficiency will contribute to the progress of HBP.

## Exogenous H_2_S in hypertension

Apart from endogenous regulation of H_2_S in HBP, exogenous administration of H_2_S would regulate the process of HBP. The effects of exogenous H_2_S donors have been widely studied in animals in different experimental settings.

NaHS was the most widely used H_2_S donor for examining the effects in treating HBP. It is reported that early treatment with sodium hydrosulfide (NaHS) (14umol/Kg/day daily intraperitoneal injection for 4 weeks) was proved to prevent the transition from pre-hypertension to hypertension in spontaneously hypertensive rats (SHRs) (24). In another study, NaHS was reported to improve endothelial dysfunction by inhibiting the NLRP3 inflammasome and oxidative stress in SHRs. However, the protective effects were abolished by knocking out Nrf2 (25). The protective effects of NaHS was also testified in an Ang-II induced HBP model, suggesting the universal effects of H_2_S in treating HBP (26). Xiao and colleagues reported that 20-week administration of NaHS lowered the arterial pressure and increased the production of NO, enhancing eNOS phosphorylation through the activation of peroxisome proliferator-activated receptor/protein kinase B/AMP-activated protein kinase (PPAR-d/Akt/AMPK) signaling pathway (27). Administration of NaHS exerted anti-hypertensive effects, promoted non-NO-mediated relaxation, and decreased oxidative stress in rats with plumbum-induced hypertension (14). Injection of NaHS was demonstrated to ameliorate soluble FMS-like tyrosine kinase 1 (sFlt1)-induced hypertension, proteinuria, and glomerular endotheliosis in rats by increasing vascular endothelial growth factor (VEGF) expression (28). NaHS administration in SHRs were proven to reduce hypertensive related inflammation, partly through regulation of T cell subsets balance by Connexin (Cx) 40/Cx43 expressions inhibition (29). These experiment results demonstrate that NaHS dramatically suppressed the progression of HBP in different experimental settings *via* different mechanisms.

GYY4137 was synthesized in 2018, characterized as a novel, water-soluble and long-releasing hydrogen sulfide-releasing molecule (30). GYY4137 has been reported to have anti-hypertensive effects, due to upregulating the expression of VEGFR2 (31). In another study, GYY4137 reversed blood pressure increase after Ang-II inducement, which was accompanied by upregulation of microRNA-129 (32). Exogenous GYY4137 supplementation in the paraventricular nucleus (PVN) attenuated sympathetic activity and hypertensive response, partly due to decrease of reactive oxygen species (ROS) and pro-inflammatory cytokines within the PVN in high salt-induced hypertension (33).

Apart from GYY4137 and NaHS, various H_2_S releasing organic compounds have been shown to exhibit protective effects in treating HBP. Allicin, which comprised a variety of sulfur-containing compounds, has been reported to exert anti-hypertensive effects in an endothelium dependent pathway (34). Sodium thiosulfate, a reversible oxidation product of H_2_S, has vasodilating and anti-oxidative properties in a N-ω-nitro-L-arginine (L-NNA) induced hypertension model (35). N-phenylthiourea (PTU) and N,N’-diphenylthiourea (DPTU) compounds have been investigated as potential H_2_S-donors, and also demonstrated typical H_2_S-mediated vascular properties (36). This experimental evidence advocates more extensive discovery of new H_2_S donors to exert more extensive application in treating HBP.

H_2_S does share interplay with NO and CO, regulating the pathogenesis of HBP. H_2_S and NO are both vasodilating mediators. H_2_S donors were reported to induce vasorelaxation and promote NO-donor induced vasorelaxation in rat thoracic aorta, showing the possible interaction between NO and H_2_S in vascular regulation (37). In _L_-NAME induced hypertensive rats a dysfunctional H_2_S pathway was revealed and exogenous H_2_S attenuated the elevated blood pressure in this model (38). Reducing CO levels in Brown-Norway rats increases H_2_S generation and prevents hypoxia-induced pulmonary edema. Increasing CO levels in SHR has been found to enhance carotid H2S generation, prevent hypersensitivity to hypoxia and control hypertension in SHR (39). H2S has also been demonstrated to exert protective effects for acute CO poisoning patients (40).

As for the mechanisms of H_2_S in regulating HBP, they share similar functions and several similar pathways to regulate hypertension. The possible mechanisms of H_2_S on vascular tone include: KATP- channel dependent relaxation, other K^+^ channels, PKG activation, hyperpolarization, eNOS inhibition, inhibition of cytochrome C oxidase and anti-oxidant effects (21, 41–47).

H_2_S not only exert anti-hypertensive effects in systematic hypertension, it also has a regulatory role in pulmonary hypertension (PHT). PHT is characterized by blood pressure increased in pulmonary artery, associated with high incidence of mortality and morbidity (48). H_2_S was reported to exert anti-hypertensive effects in pulmonary hypertension *via* vaso-relaxative actions (49, 50). H_2_S has been proven to effectively inhibit hypoxia-induced increase in cell proliferation, migration, and oxidative stress in pulmonary artery smooth muscle cells (PASMCs) in an endoplasmic reticulum (ER) -dependent pathway, therefore exerting protective effects in PHT (51). Rashid et al. demonstrated that the relaxation response of to NaHS in porcine lungs was reduced in the presence of a high concentration of K^+^, indicating that the mechanism of relaxation depends, in part, on K^+^ channel activity (52). Du group showed that H2S treatment attenuated the oxidative stress accompanied by PHT, by reducing oxidized glutathione content (53). It was also reported that endogenous sulfur dioxide pathway was down-regulated in rats with PHT, indicating the involvement of sulfur dioxide/aspartate aminotransferase 2 pathway (54).

To summarize, due to the complexity of HBP management and lack of adequate therapy, H_2_S is gaining increasingly attention as a potential therapeutic target. Therefore, we summarized the role of H_2_S in regulating hypertension in **Table 1**. However, the effects and mechanisms by which H_2_S regulates HBP are complicated and still remaining largely unknown.

**Table 1:** Role of H_2_S in Regulating Hypertension.

Source	Gene/Compound	Effects	Mechanism	Reference
Endogenous	CSE	Inhibit inflammation	Dependent on AMPK pathway	(17)
	CSE inhibitor	Increase hypertension	Not applicable	(18)
	CSE inhibitor or CBS inhibitor	Increase hypertension	Not applicable	(19)
Exogenous	NaHS	Prevent HBP	restores NO bioavailability, and blocks the RAS system in the kidney	(22)
		Prevent hypertension in SHR	improve endothelial dysfunction by inhibiting the NLRP3 inflammasome and oxidative stress	(25)
		Protective in Ang II induced HBP mice	reduces blood pressure, endothelial dysfunction and vascular oxidative stress	(26)
		Protective in HBP	increased the production of NO, enhancing eNOS phosphorylation *via* PPAR-d/Akt/AMPK pathway.	(27)
		anti-hypertensive in plumbum-induced hypertension	promoted non-NO-mediated relaxation, and decreased oxidative stress	(14)
		anti-hypertensive	Ameliorate proteinuria, and glomerular endotheliosis by increasing VEGF expression	(28)
		reduce hypertensive related inflammation	regulation of T cell subsets balance by Cx 40/Cx43 expressions inhibition	(29)
	GYY4137	anti-hypertensive	upregulating the expression of VEGF receptor 2	(31)
		anti-hypertensive after Ang-II inducement	upregulating of micro RNA-129	(32)
		attenuated sympathetic activity and hypertensive response in the paraventricular nucleus	decrease of reactive oxygen species and pro-inflammatory cytokines	(33)
	Allicin	exert anti-hypertensive effects	Dependent on endothelium	(34)
	Sodium thiosulfate	Protective in HBP	vasodilating and anti-oxidative properties	(35)
	thiourea	vasorelaxing effects	membrane hyperpolarization, mediated by activation of KATP and Kv7 potassium channels.	(36)

CSE, cystathionine-c-lyase; CBS, cystathionine-b-synthase; HBP, high blood pressure; AMPK, adenosine monophosphate activated protein kinase; RAS, renin-angiotensin system; NLPR3, Nucleotide-binding oligomerization domain, leucine-rich repeat and pyrin domain-containing 3; NO, nitric oxide; eNOS, endothelial nitric oxide synthase; Cx, Connexin; VEGF, vascular endothelial growth factor; KATP, ATP-sensitive potassium channel.

## H_2_S and atherosclerosis

Atherosclerosis (AS) is a long-term, chronic inflammatory disease of the vessel wall, which is widely recognized as a high risk for cardiovascular diseases (55). The progression of AS is extremely complex, involving numerous pathophysiological processes, including endothelial dysfunction, oxidative stress, inflammation, vascular smooth muscle cell proliferation and migration (56).

H_2_S has been reported to be a vaso-relaxant agent, which processes the property of ameliorating vascular dysfunction and mitigating the progression of AS. The potential therapeutic effects in anti-AS include maintaining endothelial cell dysfunction, inhibiting inflammation, suppressing vascular smooth muscle cell (VSMC) proliferation, migration and mitigating oxidative stress (57). However, the mechanisms of H_2_S to be protective against AS have not been fully elucidated and the therapeutic potential of H_2_S for AS treatment needs further exploration. Herein, we will review the recent findings of H_2_S in anti-AS from two main perspectives: endothelial cell dysfunction and inflammation.

### Endothelial cell dysfunction

The endothelial cell represents a fundamental barrier for the maintenance of vascular homeostasis. Dysfunction in the endothelium may lead to several cardiovascular diseases (58, 59). Therefore, protecting the vascular endothelium from damage is one of the key factors against AS and AS related disorders.

Endogenous regulation of H_2_S are observed to play a role in regulating AS. The concentration of H_2_S was found to be decreased in AS mice, indicating the potential regulatory role of H_2_S in AS (60). As mentioned previously, the synthesis of H_2_S are regulated by 3 enzymes: CBS, CSE, and 3-MST. Loss of enzyme functions may lead to endothelial dysfunction in AS. For example, CSE/H_2_S pathway is reported to involve in AS *via* the H_2_S/CSE-TXNIP-NLRP3-IL-18/IL-1β-nitric oxide (NO) signaling pathway (61). Furthermore, Tian and colleagues observed that H_2_S deficiency derived from CSE depletion contributes to the development of endothelial dysfunction. In their study, MAPK/TXNIP (thioredoxin interacting protein signaling) is positively involved in CSE/H_2_S deficiency-associated endothelial dysfunction (62). CSE/H_2_S pathway may be protective against the formation of uremia accelerated atherosclerosis (UAAS) by affecting the expression of downstream molecule endothelial nitric oxide synthase (eNOS), which may be mediated by conventional protein kinase C (PKC) βII/Akt signaling pathway (63). Besides CSE, CBS was also observed to play a role in the process of AS. Mutations in the CBS gene are known to cause endothelial dysfunction responsible for cardiovascular and neurovascular diseases, and CBS/H_2_S pathway interacts with mitochondrial function and ER-mitochondrial tethering, therefore interfering with endothelial cell dysfunction-related pathologies (64). However, the role of 3-MST in maintain endothelial cell function in AS needs to be investigated. Collectively, the level of H_2_S and CSE/CSB/3-MST level can be considered as potential biomarkers and therapeutic targets for AS patients.

Numerous studies have demonstrated that exogenous H_2_S supplementation is another source contributing to the anti-AS effects. For instance, NaHS was proved to be protective against AS by upregulating angiotensin converting enzyme 2 (ACE2) expression in endothelial cells (65). Besides, H_2_S can reverse the endothelial dysfunction induced by AngII in HUVECs by ER stress pathway (66). Furthermore, H_2_S can enhance activator protein 1 (AP-1 binding) activity with the sirtuins 3 (SIRT3) promoter, thereby upregulating SIRT3 expression and ultimately reducing oxidant-provoked vascular endothelial dysfunction (67). Also Ford reported that NaHS treatment significantly reduced endothelial dysfunction and inhibited vascular superoxide generation in high-fat diet ApoE^(-/-)^ mice, and therefore impaired atherosclerotic lesion development (68).

Apart from supplementation of traditional donors, administration of organic H_2_S donors would also be protective in maintaining EC function against AS. GYY4137 can induce autophagy and can protect ECs from Ox-LDL-induced apoptosis by activating Sirt1 (69). AP39 and AP123, the newly synthesized mitochondria-target H_2_S donors, are reported to protect endothelial cells from highglycemia-induced injury *via* preserving mitochondria function (70). With the development of pharmacy technology, the synthesis of new H_2_S releasing compounds are promising against AS.

There is crosstalk between H_2_S and NO in regulating the pathogenesis of AS. H_2_S was reported to increase NO production and upregulated the expression of inducible nitric oxide synthase(iNOS) (71). ApoE^-/-^ mice fed with PAG was found with enhanced atherosclerotic lesion area, and with decreased NO levels, suggesting H_2_S could regulate atherosclerosis progression through NO crosstalk. H_2_S partially restores aortic endothelium-dependent relaxation in ApoE^-/-^ mice, which may be related to increased phosphorylation of eNOS in the aorta (72).

To summarize, large numbers of studies have demonstrated the protective role of H_2_S in anti-AS *via* maintaining normal EC function, however, the mechanisms need deeper understanding. As a result, this will further facilitate the development of drug therapy for treating AS.

### Inflammation

AS is a chronic vascular inflammatory disease and inflammation exists at all stages of AS (73). H_2_S has been reported to have anti-inflammatory effects, further regulating the pathogenesis of AS. Deeper understating of the protective effects of H_2_S donors *via* inhibiting inflammation will help provide a new way for future AS treatment.

Endogenous H_2_S production has been reported to regulate inflammation in AS by its producing enzymes. Alterations of CSE/H_2_S pathway may thus be involved in atherosclerosis pathogenesis (74). However, the underlying mechanisms are poorly understood. Endogenous CSE/H_2_S can directly sulfhydrate SIRT1, promote its deacetylation activity, and increase SIRT1 stability, thus reducing atherosclerotic plaque formation, by reducing vascular related inflammation (75). In another study, zofenopril at, the active metabolite of zofenopril has been reported to exert anti-inflammatory activity in vascular cells through its ability to increase H_2_S availability, therefore providing a potential target for treating AS (76). Moreover, CSE/H_2_S pathway has been reported to play an anti-inflammatory role in oxidized low-density lipoprotein (ox-LDL)-stimulated macrophage by suppressing c-Jun N-terminal kinase (JNK)/NF-κB signaling pathway (74). Furthermore, high fat diet is a predisposing factor for the progression of AS. It is reported that high fat diet might cause impaired function of CSE/H_2_S pathway, aggravating inflammation and posing risks to the development of AS (77). Apart from CSE regulation pathway, it is shown that deletion of CBS would impair endogenous H_2_S production and promote inflammatory reaction in AS-susceptible mice (78). This provided evidence that H_2_S releasing diet may help protect against AS.

Apart from endogenous H_2_S in regulating AS related inflammation, exogenous H_2_S administration also had an important role in AS. NaHS was originally the most widely used H_2_S donor in studying the effects of H_2_S in anti-AS. Numerous studies have demonstrated NaHS to be protective against AS by reducing inflammation (74, 75, 79, 80). In addition to traditional H_2_S releasing salts, new synthesized H_2_S donors have shown great potential with physiological properties. Whiteman and et al. demonstrated that GYY4137 could significantly inhibit lipopolysaccharide (LPS) -induced release of pro-inflammatory mediators and promoted the release of the anti-inflammatory chemokines. While NaHS exerted a bidirectional effect at high concentrations. This finding can partly explain the complex regulation system of H_2_S in inflammation (81). GYY4137, has also been proved to be protective against the development of diabetes-accelerated AS by preventing the activation of NLPR3 inflammasome (82). Furthermore, H_2_S rich compounds are reported to upregulate the expression of glutathione (GSH) and glutamate-cysteine ligase catalytic (GCLC) subunit, inhibiting inflammation, and exerting beneficial effects of mitigating AS (83). The effects of endogenous H_2_S and exogenous H_2_S in AS were listed in **Table 2**.

**Table 2:** Effects of Endogenous H_2_S and Exogenous H_2_S in AS.

Source	Gene/Compound	Effects	Mechanism	Reference
Endogenous	CSE	CSE deficiency upregulated the levels of IL-1β and IL-18 inflammatory cytokines	*Via* activating TXNIP-NLRP3-IL-18/IL-1β-NO signaling pathway	(47)
		CSE depletion contributes to the development of endothelial dysfunction in AS	*Via* activating MAPK/TXNIP pathway	(48)
		protective against the formation of uremia accelerated atherosclerosis	*Via* activating eNOS/PKC βII/Akt signaling pathway	(49)
		reducing atherosclerotic plaque formation, by reducing vascular related inflammation	sulfhydrate SIRT1, promote its deacetylation activity, and increase SIRT1 stability	(59)
		anti-inflammatory role in ox-LDL-stimulated macrophage	suppressing JNK/NF-κB signaling pathway	(58)
Exogenous	NaHS	protective in endothelial cells	upregulating ACE2 expression	(51)
	NaHS	reverse the endothelial dysfunction induced by AngII in HUVECs	*via* ER stress pathway	(52)
	NaHS	improve vascular function by reducing vascular superoxide generation and impairing atherosclerotic lesion development	reducing endothelial dysfunction and inhibiting vascular superoxide generation	(54)
	GYY4137	reducing oxidant-provoked vascular endothelial dysfunction	upregulate activator protein 1 activity with the SIRT3 promoter	(53)
	GYY4137	protect endothelial cells from Ox-LDL-induced apoptosis by activating Sirt1	induce autophagy	(55)
	GYY4137	inhibit lipopolysaccharide -induced release of pro-inflammatory mediators and promoted the release of the anti-inflammatory chemokines	Not applicable	(65)
	GYY4137	be protective against the development of diabetes-accelerated AS	preventing the activation of NLPR3 inflammasome	(66)
	AP39 and AP123	protect endothelial cells from highglycemia-induced injury	preserving mitochondria function	(56)
	zofenoprilat	exert anti-inflammatory activity in vascular cells	In a CSE/H_2_S-mediated manner	(60)

CSE, cystathionine-c-lyase; NO, nitric oxide; TXNIP, thioredoxin-interacting protein; NLPR3, Nucleotide-binding oligomerization domain, leucine-rich repeat and pyrin domain-containing 3; NO, nitric oxide; MAPK, mitogen-activated protein kinase; eNOS, endothelial nitric oxide synthase; PKC, protein kinase C; SIRT, Sirtuin; ox-LDL, oxidized low-density lipoprotein; JNK, c-Jun N-terminal kinase; ACE2, angiotensin converting enzyme 2; ER, endoplasmic reticulum; HUVEC, human umbilical vein endothelial cell.

## H_2_S and angiogenesis

Angiogenesis is a process of new vessel formation from the existing vasculature (84). It is found that H_2_S might be a pro-angiogenic factor, promoting angiogenesis in different diseases and increase the expression of angiogenesis related biomarkers, including diabetes mellitus (DM), ischemic diseases and cancer (85).

## H_2_S and DM related angiogenesis

DM is the leading cause of mortality worldwide, causing a variety of vascular complications (86). Impaired angiogenesis is a strong feature of DM and it can commonly induce refractory wound lesions. Therefore, promoting angiogenesis is of crucial importance for DM patients.

DM patients are reported with lower concentration of H_2_S in serum and in curtenous tissues, indicating the impaired synthesis of H_2_S production in DM patients (87, 88). Therefore, regulation of endogenous H_2_S production and production enzymes are a potential treatment for DM related wound healing. CSE down-regulation is reported to play a role in the pathogenesis of diabetic impaired wound healing (89). Danhong, a traditional Chinese herb medicine, has been reported to promote angiogenesis in the diabetic hind limb ischemia model through activation of local CSE-H_2_S-vascular endothelial growth factor (VEGF) axis (90). Furthermore, DM leads to the dysfunction of 3-MST/H_2_S and 3-MST might be a therapeutic target for DM patients (91). Besides, 3-MST/H_2_S axis was also reported to exert pro-angiogeneic effects *via* modulating mitochondrial respiration and increasing mitochondrial adenosine triphosphate (ATP) production (92).

Numerous studies have proved H_2_S to be a pro-angiogenetic factor (89, 93, 94). For example, H_2_S has been reported to increase angiogenesis in injured ischemic adductor muscle and to promote the ischemic diabetic wound healing in type 2 diabetic db/db mice (95). H_2_S improves wound healing by restoration of endothelial progenitor cell (EPC) functions and activation of Ang-1 in type 2 diabetic mice (94). H_2_S can also improve diabetic impaired wound healing by attenuating inflammation and increasing angiogenesis (96). These findings together imply that H_2_S played a role in DM mediated angiogenesis.

Apart from traditional widely-know H_2_S releasing donors, new and effective donors containing H_2_S moiety have been synthesized and utilized in DM related diseases. HA-JK1 and SA/JK-1 have been synthesized as examples. For HA-JK1, an *in situ* forming biomimetic hyaluronic acid (HA) hydrogel was used as a matrix to dope a pH-controllable H_2_S donor, JK1, to form a novel HA-JK1 hybrid system. This HA-JK1 hydrogel was designed as an ideal delivery scaffold for JK1 with pH-dependent prolonged H_2_S releasing profile (97). For SA/JK-1, which was capable of releasing H_2_S consistently under acidic pH conditions by absorbing exudate at the wound interface. The SA/JK-1 sponge exhibited biocompatibility to fibroblasts and promoted cell migration *in vitro*, and exhibited obviously positive influence on wound healing, therefore providing an effective treatment for non-healing wound (98). Interestingly, microparticles containing NaHS, have been synthesized using the emulsion technique, called NaHS@MPs. It can sustainably release H_2_S under physiological conditions and promote angiogenesis, further accelerating the healing of full-thickness wounds in diabetic mice (99).

Collectively, the role of H_2_S in DM related angiogenesis is gaining increasingly attention. However, there remains large space to be explored to clinical practice.

## H_2_S and angiogenesis in ischemic diseases

Ischemic diseases are accompanied by shortage of blood supply. Angiogenesis would potentially increase the blood flow, therefore exerting the treating effects.

Modulation of endogenous H_2_S generation has a role in angiogenesis. CBS, CSE and 3-MST responded differently to angiogenesis. CSE is reported to promote VEGF-dependent angiogenesis through H_2_S generation under amino acid restriction (100). However, in another study, Tao and et al. found that CBS could promote vascular endothelial cell migration both under normoxic and minor hypoxia conditions (10% oxygen), while CSE had the opposite effects. 3-MST can accelerate the migration of endothelial cells in hypoxia, while no such effect was observed under normoxic conditions. They further found that 3-MST can modulate the endothelial cell migration, rather than CSE or CBS. Their study highlighted the need to get deeper understanding of the different functions of the H_2_S producing enzymes under different conditions (101). Furthermore, thiosulfate, one of the products formed during oxidative H_2_S metabolism, has surprisingly demonstrated inhibitory effects on VEGF-dependent endothelial cell proliferation, combined with reduction of CSE expression level (102). Therefore, the role of endogenous H_2_S on angiogenesis is controversial and requires more study to elucidate the potential mechanisms.

GYY4137 was reported to promote HHcy-mediated neoangiogenesis impairment in the ischemic hind limbs of post femoral artery ligation model *via* peroxisome proliferator-activated receptor (PPAR)-γ/VEGF axis (103). DATS, an organic polysulfide releasing H_2_S, has been demonstrated to promote angiogenesis in hindlimb ischemia *via* Akt-eNOS signaling pathway (104). Furthermore, NaHS could increase NO bioavailability and promote angiogenesis in ischemia hindlimb (105). NaHS exert proangiogeneic effect mediated by interaction between the upregulated VEGF in the skeletal muscle cells and the VEGF receptor 2 (106). In another report, NaHS exerts pro-angiogeneic effects through dependent on activation of Akt (107).

Recently, with the development of material synthesis technology, various H_2_S releasing compounds have been synthesized to enhance H_2_S releasing properties. For instance, A poly (D, L-lactic-co-glycolic acid) microparticle system that contains DATS, called DATS@MPs, possess the property of slow and long-term H_2_S release. DATS@MPs have been reported to promote therapeutic angiogenesis in an ischemic mouse limb model through activating nuclear respiratory factor 2 (Nrf2) translocation, thus providing therapeutic potential in treating ischemic diseases (108). Moreover, ZYZ-803, a novel synthetic H_2_S-NO hybrid molecule, which can slowly release H_2_S and NO, has been reported to exert pro-angiogenetic effects *via* SIRT1 dependent pathway. The pro-angiogenetic effects of H_2_S are also dependent on CSE and eNOS expression *via* cross-talk between signal transducer and activator of transcription 3 (STAT3) and Ca^2+^/CaM-dependent protein kinase II (CaMKII) activation (109, 110).

Apart from ischemic limb diseases, myocardial infarction (MI) is another serious ischemic disease, which poses great danger to people’s health. GYY4137 was reported to exert pro-angiogenic effects following MI *via* endogenous natriuretic peptide activation (111). Diallyl trisulfide, a long-lasting H_2_S donor, can mitigate left ventricular dysfunction *via* inducing angiogenesis in over-loaded heart failure (112). NaHS was reported to increase angiogenesis and improve left ventricular function after MI (113). Besides, NaHS was also reported to promote angiogenesis, and mitigating the progression of heart failure by inducing matrix metalloproteinase (MMP)-2 activation and inhibiting MMP-9 and tissue inhibitor of matrix metalloproteinase (TIMP)-3 expression (114).

Newly and novel H_2_S releasing compounds have been synthesized, with the aim to over the limitations of traditional H_2_S releasing donors. Liang and et al. developed a macromolecular H_2_S prodrug. The compound comprised of a 2-aminopyridine-5-thiocarboxamide (a small-molecule H_2_S donor) on partially oxidized alginate (ALG-CHO), to obtain the slow and continuous release of endogenous H_2_S. They further formed a stem cell-loaded conductive H_2_S-releasing hydrogel through the Schiff base reaction between ALG-CHO and gelatin. They utilized the hydrogel in treating MI, demonstrating a dramatical improvement of the cardiac functions in rats (115). Moreover, S-Propargyl-Cysteine (SPRC), a novel water-soluble modulator of endogenous H_2_S production, has been demonstrated to exhibit pro-angiogenetic effects *via* the activation of STAT3. SPRC therefore provides a novel therapeutic strategy for ischemia heart diseases (116).

H_2_S and NO shared interactions in regulating angiogenesis. Aortic rings harvested from eNOS−/− mice exhibited no microvessel outgrowth in response to NaHS, compared with wild-type controls, demonstrating that NO was essential for the pro-angiogenic effect of H_2_S. Besides, chemical inhibition of CSE attenuated NO-mediated cGMP angiogenesis (44). Apart from this, NO donors increased CSE dependent H_2_S biogenesis in a cGMP-dependent manner. Pre-treating NO donors increased CSE mRNA and protein levels in smooth muscle cells increased H_2_S production (117). Taken together, NO and H_2_S contributed mutually in regulating angiogenesis.

In summary, H_2_S plays an important role in different vascular diseases. The structure of normal artery consisted of 3 layers. The inner layer lined by a monolayer of ECs is closely contacted with blood; the middle layer composed of VSMCs is located at the complex extracellular matrix; and the outer layer of arteries is composed of mast cells, nerve endings, and microvessels. Imbalance and dysfunction of the 3 layers lead to the pathogenesis of vascular diseases, especially dysfunction of EC and SMC (7). This indicates the universal functions of H_2_S in regulating different vascular diseases. Studied have focused on the effects of H_2_S from endogenous H_2_S production and exogenous H2S administration. However, the application of H_2_S in vascular diseases is still in the basic research stage. Studies and experiments of H_2_S in treating vascular diseases are required.

Future research should focus on the role and mechanism of H_2_S and different H_2_S releasing donors in treating vascular diseases. Synthetic H_2_S donors have been developed to overcome the disadvantages of traditional H_2_S donors. They can be categorized by their class of triggering mechanisms, possessing their specific delivery system and H_2_S releasing properties (118). Continuous improvements in the interaction and crosstalk between different gas transmitters in the control of vascular diseases. Exploring the therapeutic potential in regulating vascular diseases will be promising in the near future.

## Author contributions

CZ and QL wrote the first draft. XL, RW, TG, XZ and BL provided the organization and framework of the article. KL and RC provided critical revisions. All authors contributed to the article and approved the submitted version.

## Funding

The study was supported by Natural Science Foundation of China (82000390), China Postdoctoral Science Foundation (2020M681048), and Special Fund of Jilin Province (2020SC2T038). Jilin Provincial Finance Department, China (2020SCZT091). The Jilin Science and Technology Agency funds in China, China (20210402003GH).

## Conflict of interest

The authors declare that the research was conducted in the absence of any commercial or financial relationships that could be construed as a potential conflict of interest.

## Publisher’s note

All claims expressed in this article are solely those of the authors and do not necessarily represent those of their affiliated organizations, or those of the publisher, the editors and the reviewers. Any product that may be evaluated in this article, or claim that may be made by its manufacturer, is not guaranteed or endorsed by the publisher.
